# Multi-view convolutional neural networks for automated ocular structure and tumor segmentation in retinoblastoma

**DOI:** 10.1038/s41598-021-93905-2

**Published:** 2021-07-16

**Authors:** Victor I. J. Strijbis, Christiaan M. de Bloeme, Robin W. Jansen, Hamza Kebiri, Huu-Giao Nguyen, Marcus C. de Jong, Annette C. Moll, Merixtell Bach-Cuadra, Pim de Graaf, Martijn D. Steenwijk

**Affiliations:** 1 Department of Radiology and Nuclear Medicine, Cancer Center Amsterdam, Amsterdam UMC, Vrije Universiteit Amsterdam, Amsterdam, The Netherlands; 2 Department of Anatomy and Neurosciences, Amsterdam Neuroscience, Amsterdam UMC, Vrije Universiteit Amsterdam, Amsterdam, The Netherlands; 3 Department of Radiology, Lausanne University Hospital and University of Lausanne, Lausanne, Switzerland; 4 CIBM Center for Biomedical Imaging, Geneva, Switzerland; 5 Department of Ophthalmology, Cancer Center Amsterdam, Amsterdam UMC, Vrije Universiteit Amsterdam, Amsterdam, The Netherlands

**Keywords:** Retinal diseases, Vitreous detachment, Eye cancer, Cancer, Eye diseases, Cancer, Anatomy, Medical research, Translational research, Oncology, Cancer, Engineering, Biomedical engineering, Mathematics and computing, Computational science

## Abstract

In retinoblastoma, accurate segmentation of ocular structure and tumor tissue is important when working towards personalized treatment. This retrospective study serves to evaluate the performance of multi-view convolutional neural networks (MV-CNNs) for automated eye and tumor segmentation on MRI in retinoblastoma patients. Forty retinoblastoma and 20 healthy-eyes from 30 patients were included in a train/test (N = 29 retinoblastoma-, 17 healthy-eyes) and independent validation (N = 11 retinoblastoma-, 3 healthy-eyes) set. Imaging was done using 3.0 T Fast Imaging Employing Steady-state Acquisition (FIESTA), T2-weighted and contrast-enhanced T1-weighted sequences. Sclera, vitreous humour, lens, retinal detachment and tumor were manually delineated on FIESTA images to serve as a reference standard. Volumetric and spatial performance were assessed by calculating intra-class correlation (ICC) and dice similarity coefficient (DSC). Additionally, the effects of multi-scale, sequences and data augmentation were explored. Optimal performance was obtained by using a three-level pyramid MV-CNN with FIESTA, T2 and T1c sequences and data augmentation. Eye and tumor volumetric ICC were 0.997 and 0.996, respectively. Median [Interquartile range] DSC for eye, sclera, vitreous, lens, retinal detachment and tumor were 0.965 [0.950–0.975], 0.847 [0.782–0.893], 0.975 [0.930–0.986], 0.909 [0.847–0.951], 0.828 [0.458–0.962] and 0.914 [0.852–0.958], respectively. MV-CNN can be used to obtain accurate ocular structure and tumor segmentations in retinoblastoma.

## Introduction

Retinoblastoma (RB) is the most common ocular cancer worldwide^1^ and originates from immature retinal cells in children. In RB, magnetic resonance imaging (MRI) is routinely used to confirm the diagnosis and determine disease extent^2^. In current practice, images of RB patients are assessed qualitatively to support diagnosis and gain insight into tumor extent and to assess metastatic risk factors^3–5^. Related work in other cancers has shown that quantitative assessment of radiological features (i.e., radiomics) may provide additional insights into tumor characteristics and harbor predictive and prognostic information^6^. MRI-based radiomic models have for instance been proposed for the head-and-neck site^7,8^ and uveal melanoma (UM)^9^.

Current application of radiomics in RB is limited due to the time-consuming and subjective procedure of manual delineation that is necessary for obtaining tissue segmentations^10^. In addition, automated segmentation of MR images in RB is challenging due to data scarcity, images being acquired under different conditions and large variability in terms of pathology^11^. Regardless, some methods have been proposed for the automation of ocular structure and tumor segmentation^10–16^. Traditionally, ocular structure and tumor segmentation is performed by using Active Shape Models (ASMs)^12–15^ in combination with 2D or 3D U-Nets^11,14–16^. Important limitations of ASM and sequential segmentation designs are the need for feature engineering and limited predictability of the algorithm in the presence of tumor tissue. U-nets, on the other hand, have the disadvantage of a limited field of view (in the 2D-case)^16^ or do require an extraordinary amount of data (in the 3D-case)^11^ for proper training.

A one-step solution for segmentation of ocular and tumor structures would greatly simplify the use of radiomics in the clinic and research and, importantly, also has the potential of increasing accuracy. Therefore, the purpose of the current work is to evaluate the performance of multi-view convolutional neural networks (MV-CNN) in RB patients. MV-CNN has been successfully applied in other medical segmentation problems where relatively little data was available (e.g. MS lesions^17,18^ lymph nodes^19^) and is likely to be robust even in a longitudinal manner^20^. In contrast to above-mentioned ASM-based models, MV-CNN allows for multi-class segmentation of healthy and pathological ocular regions in a single step, without the need for feature engineering. Specifically, we train the classifier to discriminate background, sclera, vitreous humour, lens, retinal detachment and tumor. Throughout this work, the retinal detachment is regarded as retina and sub-retinal fluid resulting from retinal detachment. We compared our results to an established ‘baseline’ model published in literature.

## Results

Manually segmented volumes of healthy and RB eyes were on average 5.82 ± 1.22 mL and 5.46 ± 1.17 mL respectively. Median [interquartile range (IQR)] manual tumor volume was 0.88 [0.53–1.61] mL. An overview of performances for different MV-CNN model alterations is given in Supplementary Table S1. The MV-CNN model that used all sequences (Fast Imaging Employing Steady-state Acquisition; FIESTA, T2 and T1c) and multi-scale information showed the highest volumetric and spatial performance in 4 out of 6 classes. This MV-CNN configuration was regarded as best performing model and is further reported below.

For the best performing MV-CNN model, healthy and RB eye volumes were on average 6.15 ± 1.27 mL and 5.82 ± 1.22 mL, respectively, and the median [IQR] tumor volume was 0.97 [0.54–1.56] mL. Inference took at maximum 20 seconds per eye. Three example segmentations are shown in Fig. 2. Compared to the reference segmentations, MV-CNN reached very high volumetric performance (ICC > 0.99 for both eye and tumor volume; see Table 1 and Fig. 3) and good spatial performance (mean ± sd, median [IQR] DSC of 0.97 ± 0.01, 0.97 [0.95–0.98] and 0.85 ± 0.23, 0.91 [0.85–0.96] for eye and tumor, respectively). Compared to the previously published state-of-the-art baseline model, which relies on a sequential pipeline combining ASM and a 2D U-Net, MV-CNN showed better volumetric performance for both eye and tumor volume and spatial performance improved significantly for both eye and tumor segmentations (both p < 0.002; Table 1). Axial- and 3D-view networks differed significantly (both p < 0.01) from MV-CNN for the tumor case with DSCs of 0.78 ± 0.22, 0.84[0.78–0.89] and 0.78 ± 0.22, 0.83[0.79–0.88], respectively, and showed inferior performance for all classes and inferior or comparable performance for complete eye. Boxplots containing the complete segmentation distributions of all structures using multi-view, axial-view and 3D-view CNNs can be found in Supplementary Figs. S2 and S3.

**Table 1:** Overview of volumetric and spatial performance of the baseline and the proposed multi-view convolutional neural network models, and results of the Wilcoxon signed rank test comparing baseline with MV-CNN DSC.

Structure	Baseline	MV-CNN	Baseline vs	MV-CNN
		Cross-validation (N = 46)	MV-CNN p-value	Independent validation (N = 14)
**ICC**				
Eye	0.92	0.997		0.96
Tumor	0.69	0.996		0.97
**DSC**				
Eye	0.95 ± 0.02	0.97 ± 0.01	< 0.001	0.94 ± 0.04
Sclera	0.67 ± 0.05	0.84 ± 0.03	< 0.001	0.87 ± 0.04
VH	0.79 ± 0.22	0.93 ± 0.20	0.005	0.75 ± 0.33
Lens	0.94 ± 0.02	0.91 ± 0.02	< 0.001	0.86 ± 0.06
RD	0.50 ± 0.27	0.79 ± 0.17	< 0.001	0.64 ± 0.30
Tumor	0.66 ± 0.24	0.84 ± 0.23	0.002	0.78 ± 0.25

P-values were corrected for multiple comparisons. Small, medium and large columns refer to tumor size subgroups.

*ICC* intra-class correlation; *DSC* dice similarity coefficient; *MV-CNN* multi-view convolutional neural network; *VH* vitreous humour; *RD* retinal detachment.

### Tumor size dependency

Terciles were used to group results into small (< 0.55 mL; N = 10), medium (> 0.55 mL and < 1.51 mL; N = 9) and large tumors (> 1.51 mL; N = 10). Analysis of these groups showed an average MV-CNN spatial performance of DSC = 0.72 ± 0.36, 0.90 ± 0.04 and 0.92 ± 0.02, respectively. Two very small tumors with a volume of < 0.1 mL were completely missed by the MV-CNN network.

### Independent validation set

In the independent validation set, manually segmented healthy and RB eye volume were on average 5.30 ± 1.08 mL and 4.02 ± 0.87 mL, respectively. Median [IQR] manual tumor volume was 0.87 [0.27–1.04] mL. The MV-CNN model ICC reached 0.96 and 0.97 for eye and tumor volume, respectively. Spatial performance for eye and tumor was on average DSC = 0.94 ± 0.04 and DSC = 0.78 ± 0.25, respectively; see Table 1.

## Discussion

The purpose of the current work was to evaluate the performance of MV-CNN to provide a one-step solution for segmentation of ocular structures and tumor tissue on MR images in RB patients. MV-CNN displayed good volumetric and spatial performance of MV-CNN when compared to manual reference segmentations and an established automated segmentation methodology. These findings were confirmed in an independent validation sample, underlining the practical usability of the approach for automatic delineation and incorporation in a radiomics pipeline.

Our study demonstrated that MV-CNN provides tumor segmentations that have very high volumetric (ICC > 0.99) and spatial consistency (DSC > 0.8) compared with manual delineations. Comparing our tumor segmentation results to other publications should be done with care, since measured performance is highly dependent on the dataset and the reference used for validation. Factors known to influence segmentation performance include the pulse-sequence used, construction of the reference dataset and overall burden. In addition, definition of anatomical regions can be an issue and some studies using other class definitions as compared to the current work. Taking these considerations into account, an overview of ocular segmentation literature is provided in Table 2. Bach Cuadra et al.^21^ achieved moderate to high sclera and lens segmentation performance using a parametric model on computed tomography (CT) and ultrasound (US) images of the eye to improve external beam radiotherapy (EBRT) planning for RB. Rüegsegger et al.^13^ used an ASM on adult head CT data to further improve segmentation of the eye and lens for RB EBRT planning. Comparing these works with our results is not straightforward for two reasons. First, they were done for the purpose of radiotherapy planning in which safety margins are used depending on the location of the tumor, and thus different boundary criteria and evaluation criteria may be used. Second, these studies used CT for segmentation which has less soft tissue contrast than MRI used in our study. More recent studies constructed segmentations on MRI, for example Ciller et al.^12^ and Nguyen et al.^15^ used ASM segmentation of healthy ocular structures (sclera, vitreous humour and lens average DSC = 0.949, 0.947 and 0.882, respectively).

**Table 2:** Literature overview of eye and tumor segmentation methods and performances.

Reference	Model used	Data set	Pulse sequence	Performance (DSC)			
				Sclera	VH	Lens	Tumor
Current	MV-CNN	MR, 29 RB 17 healthy eyes	T1c, T1, T2, FIESTA	0.84	0.93	0.91	0.84
De Graaf 2019^14^	ASM + 3D U-Net	MR, 24 RB, 11 healthy eyes	T2	0.90*	–	0.81	0.65
Nguyen 2019^16^	2D U-Net + ASM / CRF	MR, 24 UM	T1, T2	–	–	–	0.84
Nguyen 2018^15^	3D ASM	MR, 7 UM, 30 healthy eyes	T1	0.95*	0.92	0.88	-
Nguyen 2018^11^	3D U-Net	MR, 32 RB eyes, 40 healthy eyes^**+**^, multi-center	T1, T2	0.95*	–	0.87	0.59
Ciller 2017^10^	3D ASM + 3D CNN	MR, 16 RB eyes	3D T1c, T1, T2	0.95*	0.95	0.86	0.62
Ciller 2015^12^	3D ASM	MR, 24 healthy eyes	3D T1c	0.95*	0.95	0.85	–
Beenakker 2015^32^	Topo-graphic map	MR, 17 healthy eyes	3D IR TGE	No DSC reported			
Rüegsegger 2012^13^	3D ASM	CT, 17 healthy eyes	Does not apply	0.95*	–	0.91	–
Bach Cuadra 2010^21^	3D parametric model	US, CT, 3 RB eyes	Does not apply	0.91*	-	0.77	-

*DSC* dice similarity coefficient; *VH* vitreous humour; *MV* multi-view; *CNN* convolutional neural networks; *MR* magnetic resonance; *ASM* active shape model; *CRF* conditional random field; *UM* uveal melanoma; *RB* retinoblastoma; *IR* inversion recovery; *TGE* turbo gradient echo; *CT* computed tomography; *US* ultrasound; *T1c* T1 with gadolinium contrast.

*Includes vitreous humour.

^**+**^Includes child and adult scans.

Only four studies used deep learning methods based on CNN and U-Net architectures to segment healthy and tumor ocular tissue. First, Ciller et al. expanded their ASM method with an input for an 8-layer 3D CNN to also obtain tumor tissue^10^. At the time, this method served as a new state-of-the-art because it resulted in tumor segmentation performances up to DSC = 0.62. A weak point of the method is however that it depended on two steps requiring feature engineering, as tumor-specific features are used as input for the CNN. Second, Nguyen et al.^11^ proposed a single-step 3D U-Net CNN to achieve a reported tumor DSC of 0.59. Third, De Graaf et al.^14^ used an ASM as input for a 2D U-Net CNN to segment healthy ocular structures and tumor with DSC = 0.64, respectively. Fourth, Nguyen et al.^16^ explored a weakly supervised approach based on class activation maps to train a 2D U-Net CNN to segment UMs in 24 patients with on average DSC = 0.84. However, these methods still use post-processing steps^11^, or need an ASM to provide prior knowledge of the inside eye volume^12–14^.

Compared to the previously discussed methods, MV-CNN shows superior spatial performance in tumor segmentation, and similar performance in vitreous humour and lens segmentation. A comparison for sclera segmentation performance is unfortunately less straight forward, because in previous works it was common practice to define sclera as the sum of sclera and vitreous humour. This resulted in considerably larger sclera volumes which positively biased DSC as a performance metric^18^. Considering that the size of the sclera segmentation volume in our definition is almost twice as small compared to the former papers, we argue that the average spatial performance of DSC = 0.84 in our work was very high.

To overcome the difficulties in comparing performance metrics between studies (e.g. due to differences in data set, manual segmentation quality, or class definition), we also compared our results with an established baseline model^12,15^. This direct comparison demonstrated substantial increases of volumetric and spatial performance for almost all tissue classes except lens. This ruled out the possibility that our data set or manual reference segmentation biased the results.

Several factors may contribute to the superior performance of the MV-CNN network topology. First, the number of parameters versus the number of training samples is more efficient in a 2.5D versus 3D network, which can be beneficial in the presence of limited training data. Secondly, it is believed that the branched architecture of MV-CNN can more effectively learn and propagate higher-level features, when compared to a U-Net architecture. This is because during the down-sampling procedure, details specific to informative branches can vanish when mixed with less informative branches^18^. Finally, MV-CNN uses a multi-scale pyramid representation to integrate contextual information in the segmentation verdict. This is important because it can be argued that anatomical information within the direct vicinity of a query voxel can be of great descriptive value, resolving local ambiguities (e.g. it is unlikely that tumor is detected in or near the lens)^22^. Integration of contextual information is therefore likely to enhance model performance.

During evaluation, we also noticed some issues that may be improved in future work. First, we observed that the ASM segmentations showed generally higher spatial agreement of the lens with the manual reference compared with the MV-CNN. This can probably be explained by the fact that the ASM is superior in dealing with structures that have little shape variability among subjects. Second, we observed that MV-CNN has the tendency of a slight, but systematic, over-estimate of the total eye volume (see Fig. 3). Post-hoc investigation of the segmentation masks showed that this phenomenon is most likely driven by overestimation of the sclera. Three explanations may account for this. First, the manual annotation protocol was very conservative in this area. This may have led to a less optimal ground truth at the edges of the eye. Second, the effect could have been caused by an interpolation artefact due to the 2.5D nature of our kernel. And third, the issue might have been caused by the fact that our loss function was non-weighted. Future studies may resolve the issue by using a 3D kernel at the border or class-weighting.

Our work also has a number of limitations. First, we used very high-quality data (e.g. both in terms of image quality and labels) from only a single scanner for training and evaluation. The current method would require training for every new scanner, which is not practical. Real world applications should be able to handle data from multiple sources, especially in a rare disease such as retinoblastoma. Future work should therefore invest in multi-center labelled data and methods that are able to handle real-world scanner diversity. Second, we did not extensively investigate the effects of class imbalance and loss function. Such class imbalance is intrinsically present in data where malignant tissue is one of the target classes, and may be handled better by other loss functions such as generalized dice or boundary loss^23,24^. Future studies may investigate whether even better performance can be achieved by tuning these aspects. Finally, we did not investigate different network topologies within the MV-CNN branches themselves. It is known that the conventional double convolutional layer may be affected by loss of gradient. This is not the case with several other designs, such as ResNet^25^. Future work may investigate whether alternative branch topologies lead to even better performance.

In conclusion, we validated a multi-view convolutional neural network for automated, single-step segmentation of ocular and pathological structures for MRI in RB, and compared its performance to the current state-of-the-art. The MV-CNN model demonstrated superior performance when compared to the baseline model, both in terms of volumetric and spatial performance. In addition, we demonstrated the benefit of multi-view networks over axial-view and 3D-view networks for ocular structure and tumor segmentation in retinoblastoma. Our results indicate that MV-CNNs have great potential for further development towards automated segmentation for radiomics applications.

## Materials and methods

### Clinical dataset

The dataset consisted of N = 23 children (mean age 23.9 ± 20.7 months, range [0–75] months), with a total of 17 healthy and 29 RB eyes. MR imaging was performed on a 3.0T system (Discovery 750, GE Medical Systems, Milwaukee, USA) with a 32-channel phased-array head coil. The standard care protocol included a 3D FIESTA (TR = 8.1 ms, TE = 3.5 ms, flip angle (FA) = 40°, Field-of-view (FOV) = 140 mm, 0.27 × 0.27 × 0.30 mm^3^), a 2D T2-weighted (TR = 2980 ms, TE = 9.0 ms, FOV = 140 mm, 0.27 × 0.27 × 2.0 mm^3^) and a 2D gadolinium contrast-enhanced T1-weighted (T1c; TR = 747 ms, TE = 12.0 ms, FOV = 140 mm, 0.14 × 0.14 × 2.0 mm^3^) sequence. 2D images were acquired in axial plane according to published imaging guidelines^2^.

### Manual reference segmentation

Reference segmentations of ocular structures (sclera, vitreous humour, lens, retinal detachment) and tumor were manually constructed on the 3D FIESTA images by one rater (CdB) using 3D Slicer (Version 4.10.1, MIT, USA)^26^. All reference segmentations were validated by a neuro-radiologist with 14 years of experience (PdG). Manual segmentations were carefully constructed in approximately 10 hours per eye and were considered as ground truth in the analyses.

### Image preprocessing

Prior to automatic segmentation, images were automatically preprocessed using tools from the Insight Toolkit (ITK; https://itk.org/) and FMRIB Software Library (FSL; https://fsl.fmrib.ox.ac.uk/). First, a rough outline of the eye was constructed on each sequence by using the 3D Hough filtering approach implemented in ITK^27^. These masks, extrapolated by a radius of 25 mm, were used as a region of interest for co-registration of the images of a specific subject. The rigid transformations between high-resolution FIESTA and lower resolution T2 and T1c space were obtained using FSL FLIRT. Both transformation matrices were inverted to obtain all 2D sequences in 3D FIESTA space using spline interpolation. Finally, the intensities of each contrast were re-scaled such that image intensities had a mean and variance of 0 and 1, respectively, within the union of 5-mm masks of the left and right eye.

### Baseline model

We have combined two state-of-the-art methods to act as reference model for comparison with the MV-CNN approach. In summary, the ASM approach previously used^12,15^ was retrained on the FIESTA images to segment the sclera, lens and vitreous humour. Subsequently, adopting recent ocular tumor segmentation methods, a 2D U-Net architecture was trained to obtain tumor and retinal detachment masks using the combined FIESTA, T2 and contrast-enhanced T1 as inputs^14,16^. We refer the reader to the Supplementary material for details on 2D U-Net implementation and to Supplementary Fig. S1 online for a schematic representation of the baseline model.

These state-of-the art methods for healthy and pathological structures proceed to each structure segmentation separately and as such they need afterwards to combine their outputs to assign one class per voxel. Similarly to previous studies^11,13^, tumor and retinal detachment predictions were constrained to be inside the eye as defined by ASM output of sclera. Moreover, lens was prioritized over retinal detachment and tumor, and retinal detachment was prioritized over tumor. Finally, as ASM segmentation is based on the structure outer contour, the output sclera and vitreous humour are converted to binary masks that include all voxels inside their fitted contours^14,15^. Scleral segmentation is obtained by removing all other structures’ subsets, and finally vitreous humour is obtained by removing retinal detachment and tumor from the ASM segmentation.

### Multi-view convolutional neural network

**Figure 1:**
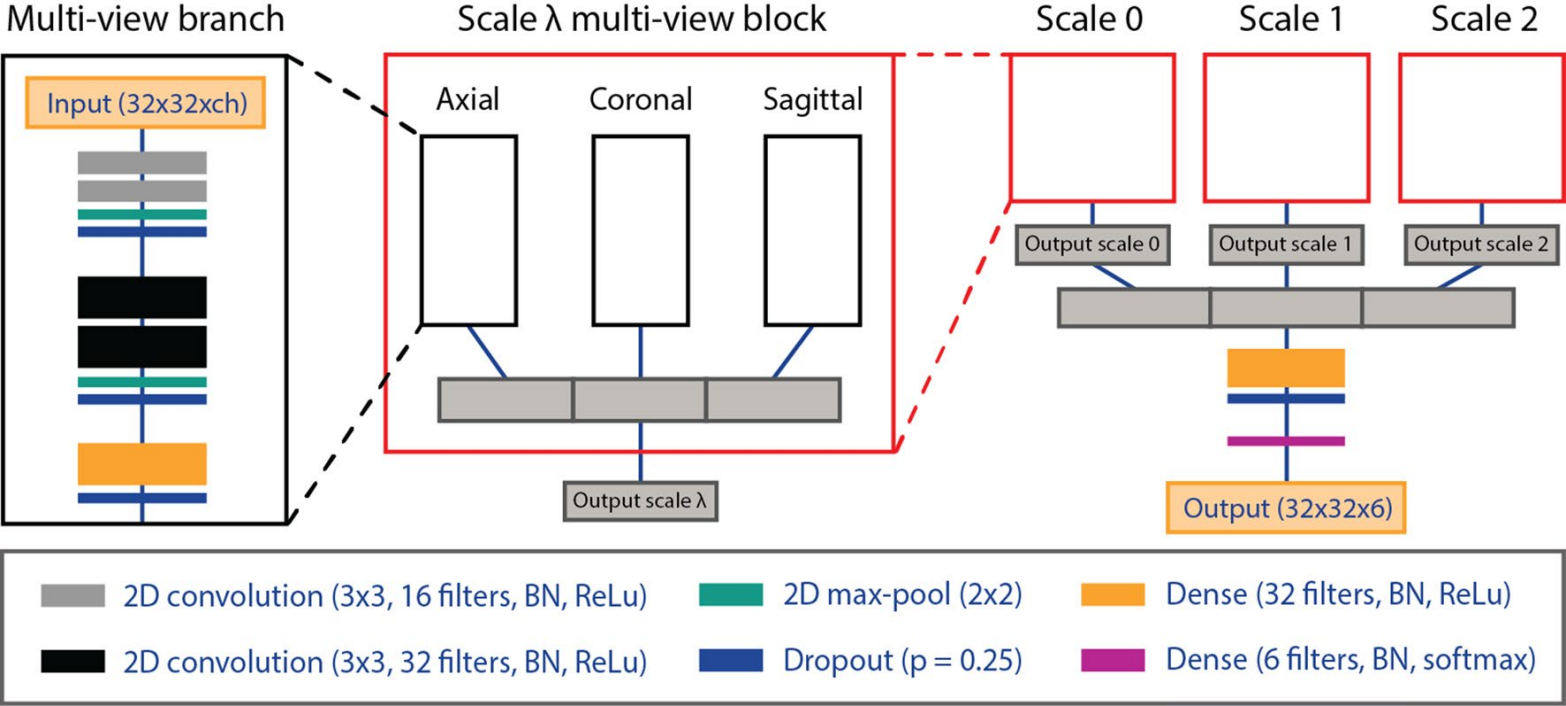
Schematic representation of the multi-view convolutional neural network (MV-CNN) architecture. Three multi-view branches build up each anatomical plane within a scale block. The output of which is concatenated and used as input for the multi-scale branched architecture. Batch normalization is always applied before the non-linear activation function. Thickness of convolutional and dense blocks correspond with the number of filters used. *ch* number of channels; *λ* scale; *BN* batch normalization; *ReLu* rectified linear unit. Figure was generated with Adobe Illustrator (version 16.0.0; https://www.adobe.com/creativecloud.html).

MV-CNN is a network topology that combines information from different views into fully connected layers to classify the voxel where the planes cross. The multi-view approach (see Fig. 1) can be considered as a 2.5D CNN given that it incorporates information from each image plane, but does not use the full 3D neighborhood of the queried voxel. This results in a lower computational complexity when compared to 3D-kernel methods. Multi-scale contextual information is incorporated from different scales λ in a pyramid representation of each patch. Increasing image scale beyond scale 3 was investigated but did not improve segmentation accuracy as the field of view would simply fall outside of the region of interest. One MV-CNN block contains three equally structured network branches for each imaging plane. The input to each branch is a 32 × 32 patch from each MR sequence (FIESTA, T2, T1c; total number of sequences ch = 2 or 3), which are fed as channels, and scales 0, 1 and 2. Here, scale 0 refers to an unaltered patch with no larger-scale contextual information, where the considered scale’s reception field is widened by a factor of two for each subsequent pyramid level. Batch normalization is always applied before applying the activation function. Each branch contains two hidden convolutional layers (3 × 3 convolution kernel; activation function: rectified linear unit (ReLu)) with a max-pool layer (2 × 2 max-pool kernel) and a dropout layer (dropout proportion p = 0.25). This is followed by a dense layer with 32 output neurons (activation function: ReLu). The results from each anatomical plane are then concatenated and the procedure is repeated for each scale in parallel. In a similar fashion, results from each scale are then concatenated. Following, another dense layer with 32 output neurons (activation function: ReLu) is used with a dropout layer (p = 0.25). Finally, a dense layer (activation function: softmax) is used for voxel classification.

#### Experiments

FIESTA and T1c images were used as input for every experiment. In addition, we investigated the effects of the following alterations: (i) addition of T2 to the input; (ii) addition of multi-scale information (i.e., λ = 1,2); and (iii) random left–right mirroring of the input data to facilitate data-augmentation, resulting in 8 configurations. Each sub-model was trained once for performing multi-class segmentation in one step. Performances were evaluated by leave-one-subject out cross-validating each possible configuration. To further demonstrate the benefits of MV-CNN, post-hoc analyses were done including axial-view and 3D-view networks. Here, the exact same settings and architecture were used as the proposed best MV-CNN sub-model (inclusion of T2, multi-scale (λ = 2), no left/right mirroring), but differed only at input-level (axial-view: one 32 × 32 view-branch and three context branches; 3D: one 32 × 32 × 32 view-branch and three context branches).

#### Model training

MV-CNN training was done on a NVIDIA GeForce GTX 1080 TI graphics processor unit (GPU) using the GPU-version of TensorFlow version 1.9.0 with Cuda 9.0 and Python 3.6.9. TensorBoard (Version 0.4.0) callback was used for tracking training and validation scores. Categorical cross-entropy was used as a loss function for multi-class segmentation:

1 $$H\left( {p,q} \right) = ~ - \mathop \sum \limits_{{c = 1}}^{C} \mathop \sum \limits_{{a = 1}}^{A} p\left( {a,c} \right)\log \left( {q\left( {a,c} \right)} \right)$$

Here, p(a) represents a reference distribution of $a \in A~$ given by the manual annotations, where q(a) is a query distribution and A is a set of observations. $c \in \left[ {0,1, \ldots ,~C} \right]$ denotes class indices. The loss function was minimized for 50 epochs (batch size = 64) using the ADAM optimizer^28^. A random sub-set of 5% of all training voxels was sampled to reduce computational demand and thereby accelerate training, and random reshuffling of samples was done to allow for varied training. Dropout was switched off at test time.

### Statistical analysis of model performance

The performance of each MV-CNN model and the baseline were assessed using leave-one-subject-out cross-validation (i.e., K-fold cross-validation, where N = 23 subjects). Performance was measured by quantifying volumetric and spatial agreement. Volumetric agreement was quantified by calculating the intra-class correlation coefficient (ICC; single measure and absolute agreement^29^) and spatial agreement was quantified by calculating DSC:

2 $$DSC = \frac{{2 \cdot \left| {A~ \cap ~B} \right|}}{{\left| A \right| + \left| B \right|}}$$

where A and B are sets that refer to the manual reference and segmentation of interest, respectively. Because DSC measures were not normally distributed upon histogram inspection, differences in spatial performance were evaluated by a two-sided Wilcoxon rank signed test. Bonferroni correction was applied to account for multiple comparisons. Since the DSC spatial performance measure is dependent on size of the underlying burden^30^, additionally, spatial performance was grouped according to tumor size.

### Independent validation set

The MV-CNN model reaching best performance was additionally evaluated in an independent validation set that consisted of 7 subjects (mean age: 16.0 ± 17.8 months, range [1–56] months), with 3 healthy and 11 RB eyes. The images of these subjects were acquired on the same scanner and using the same imaging protocols as specified above.

### Figure generation

**Figure 2:**
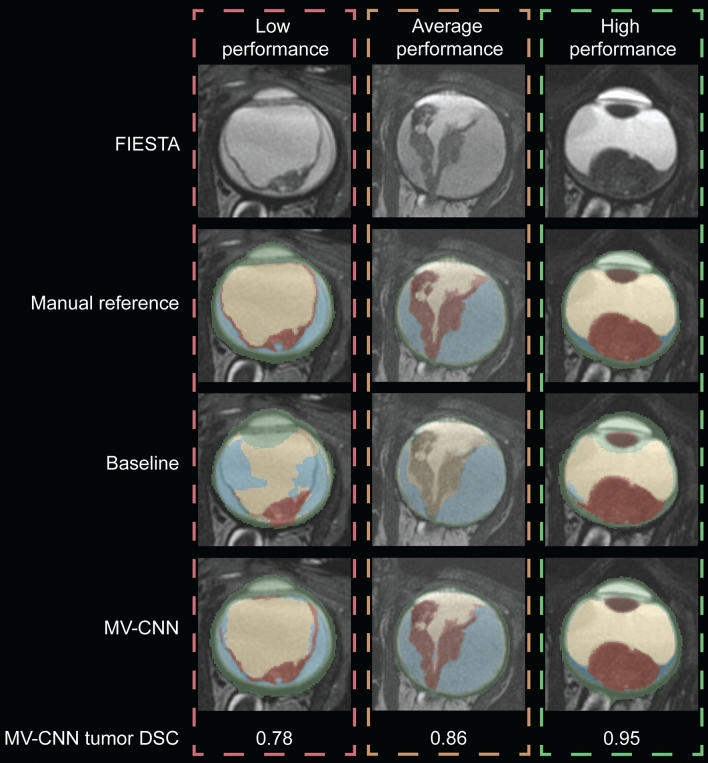
Example segmentations with relatively low (left), average (middle) and high (right) MV-CNN tumor spatial performance. Segmentation color coding: green: sclera, yellow: vitreous humour, brown: lens, blue: retinal detachment, red: tumor. *FIESTA* Fast Imaging Employing Steady-state Acquisition; *DSC* Dice’s Similarity Coefficient; *MV-CNN* multi-view convolutional neural network. Figure was generated with Adobe Illustrator (version 16.0.0; https://www.adobe.com/creativecloud.html).

**Figure 3:**
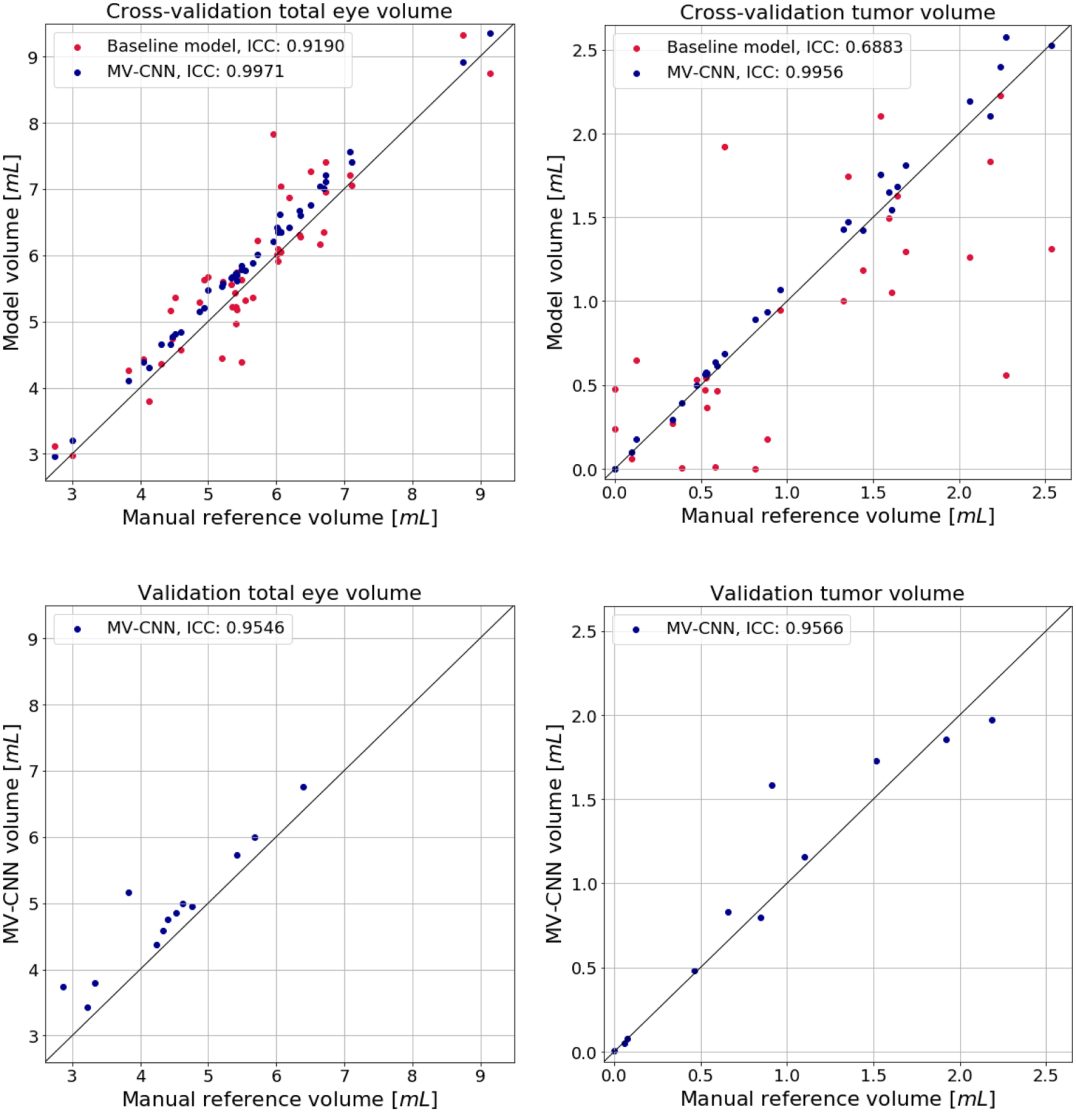
Eye and tumor volumes in the cross-validation (top) and validation (bottom) datasets. The black diagonal lines indicate identity. *ICC* intra-class correlation (single measures, absolute agreement); *MV* multi-view; *CNN* convolutional neural network. Figure was generated using Python (version 3.6.9; https://www.python.org) including the package Matplotlib (version 3.3.1; http://matplotlib.org)^31^.

Figures 1 and 2 and Supplementary Fig. S1 were generated using Adobe Illustrator (version 16.0.0; https://www.adobe.com/creativecloud.html), and Fig. 3 and Supplementary Figs. S2 and S3 were generated using Python (version 3.6.9; https://www.python.org) including the package Matplotlib (version 3.3.1; https://matplotlib.org/)^31^, by VIJS and RWJ.

### Ethical approval

All experiments in this study were performed in accordance with ethical guidelines and regulations and have been approved by the medical ethical review committee of Amsterdam UMC, location VUmc. Informed consent was given by all participants’ legal guardians.

## Supplementary Information

Supplementary Information.

## Data Availability

The datasets generated during and/or analyzed during the current study are available from the corresponding author upon reasonable request.

## References

[1] Kivelä T (2009). The epidemiological challenge of the most frequent eye cancer: Retinoblastoma, an issue of birth and death. Br. J. Ophthalmol..

[2] de Graaf P (2012). Guidelines for imaging retinoblastoma: Imaging principles and MRI standardization. Pediatr. Radiol..

[3] De Jong MC (2016). Diagnostic accuracy of intraocular tumor size measured with MR imaging in the prediction of postlaminar optic nerve invasion and massive choroidal invasion of retinoblastoma. Radiology.

[4] Jansen RW (2018). MR imaging features of retinoblastoma: Association with gene expression profiles. Radiology.

[5] Sirin S (2015). High-resolution MRI using orbit surface coils for the evaluation of metastatic risk factors in 143 children with retinoblastoma. Neuroradiology.

[6] Gillies RJ, Kinahan PE, Hricak H (2016). Radiomics: Images are more than pictures, they are data. Radiology.

[7] Mes SW (2020). Outcome prediction of head and neck squamous cell carcinoma by MRI radiomic signatures. Eur. Radiol..

[8] Martens RM (2019). Predictive value of quantitative diffusion-weighted imaging and 18-F-FDG-PET in head and neck squamous cell carcinoma treated by (chemo)radiotherapy. Eur. J. Radiol..

[9] Su Y (2020). Value of MR-based radiomics in differentiating uveal melanoma from other intraocular masses in adults. Eur. J. Radiol..

[10] Ciller C (2017). Multi-channel MRI segmentation of eye structures and tumors using patient-specific features. PLoS ONE.

[11] Nguyen, H.-G. *et al.* Ocular Structures Segmentation from Multi-sequences MRI Using 3D Unet with Fully Connected CRFs. In *1st International Workshop on Computational Pathology (COMPAY)/5th International Workshop on Ophthalmic Medical Image Analysis (OMIA)* 167–75. 10.1007/978-3-030-00949-6_20 (2018).

[12] Ciller C (2015). Automatic segmentation of the eye in 3D magnetic resonance imaging: A novel statistical shape model for treatment planning of retinoblastoma. Int. J. Radiat. Oncol..

[13] Rüegsegger MB (2012). Statistical modeling of the eye for multimodal treatment planning for external beam radiation therapy of intraocular tumors. Int. J. Radiat. Oncol. Biol. Phys..

[14] de Graaf, P. *et al.* Automated segmentation of eye structures and retinoblastoma on MRI using Unet with statistical shape priors. In *ECR 2019, Vienna, AUSTRIA*. 10.1371/journal.pone.0173900 (2019).

[15] Nguyen H-G (2018). Personalized anatomic eye model from T1-weighted volume interpolated gradient echo magnetic resonance imaging of patients with uveal melanoma. Int. J. Radiat. Oncol. Biol. Phys..

[16] Nguyen, H.-G. *et al.* A novel segmentation framework for uveal melanoma in magnetic resonance imaging based on class activation maps. In *MIDL* 370–379. 10.7892/boris.135253 (2019).

[17] Steenwijk, M. D., Daams, M., Barkhof, F., Pouwels, P. J. W. & Geurts, J. J. G. Multi-view convolutional neural networks using batch normalization outperform human raters during automatic white matter lesion segmentation. *ECTRIMS* (2017).

[18] Aslani S (2019). Multi-branch convolutional neural network for multiple sclerosis lesion segmentation. Neuroimage.

[19] Roth, H. R. *et al.* A New 2.5D Representation for Lymph Node Detection Using Random Sets of Deep Convolutional Neural Network Observations. In *Medical Image Computing and Computer-Assisted Intervention (MICCAI)* 520–27. 10.1007/978-3-319-10404-1_65 (2014). 10.1007/978-3-319-10404-1_65 PMC4295635 25333158

[20] Birenbaum A, Greenspan H (2017). Multi-view longitudinal CNN for multiple sclerosis lesion segmentation. Eng. Appl. Artif. Intell..

[21] Cuadra MB (2011). Model-Based Segmentation and Fusion of 3D Computed Tomography and 3D Ultrasound of the Eye for Radiotherapy Planning.

[22] Ding P, Zhang J, Zhou H, Zou X, Wang M (2020). Pyramid context learning for object detection. J. Supercomput..

[23] Sudre CH, Li W, Vercauteren T, Ourselin S, Cardoso MJ (2017). Generalised Dice Overlap as a Deep Learning Loss Function for Highly Unbalanced Segmentations.

[24] Kervadec, H. *et al.* Boundary loss for highly unbalanced segmentation. (2018). 10.1016/j.media.2020.101851 33080507

[25] He K, Zhang X, Ren S, Sun J (2016). Identity Mappings in Deep Residual Networks.

[26] Fedorov A (2012). 3D Slicer as an image computing platform for the Quantitative Imaging Network. Magn. Reson. Imaging.

[27] Mosaliganti, K., Gelas, A., Cowgill, P. & Megason, S. An optimized N-dimensional Hough filter for detecting spherical image objects. *Insight J.* (2009).

[28] Kingma, D. P. & Ba, J. Adam: A method for stochastic optimization. In *3rd International Conference of Learning Representations (ICLR)* (2015).

[29] Koo TK, Li MY (2016). A guideline of selecting and reporting intraclass correlation coefficients for reliability research. J. Chiropr. Med..

[30] Wack DS (2012). Improved assessment of multiple sclerosis lesion segmentation agreement via detection and outline error estimates. BMC Med. Imaging.

[31] Hunter JD (2007). Matplotlib: A 2D graphics environment, computing in science & engineering. Comput. Sci. Eng..

[32] Beenakker J-WM, Shamonin DP, Webb AG, Luyten GPM, Stoel BC (2015). Automated retinal topographic maps measured with magnetic resonance imaging. Invest. Ophthalmol. Vis. Sci..

