# Supplementary material for: Multi-view convolutional neural networks for automated ocular structure and tumor segmentation in retinoblastoma
Source: Sci Rep. 2021 Jul 16;11:14590. doi: 10.1038/s41598-021-93905-2 (PMC8285489; doi:10.1038/s41598-021-93905-2)
Supplement: Supplementary file 1 — Supplementary Information. [file 41598_2021_93905_MOESM1_ESM.docx]

**Multi-view convolutional neural networks for automated ocular structure and tumor segmentation in retinoblastoma**

Victor I.J. Strijbis MSc, Christiaan M. de Bloeme MD, Robin W. Jansen MD, Hamza Kebiri MSc, Huu-Giao Nguyen PhD, Marcus C. de Jong MD PhD, Annette C. Moll MD PhD, Merixtell Bach-Cuadra PhD, Pim de Graaf MD PhD, Martijn D. Steenwijk PhD

**SUPPLEMENTARY MATERIALS**

**Supplementary methods for the baseline model**

Based on most recent ocular tumor segmentation methods^1,2^ we have chosen to segment retinoblastoma and retinal detachment with a 2D-UNET architecture. Our 2D U-Net (see Supplementary Figure 1) was trained using the combined FIESTA, T2 and contrast-enhanced T1 as inputs. We extracted 2D patches from the three orthogonal planes of each 3D image. Patches are centered in the center of mass of the binarized masks and are 96x96 in-plane, and 72x96 in the two remaining planes. The three orthogonal planes were used for training whereas testing was performed in the acquisition plane. Extensive data augmentation was performed on the fly: flipping, rotating, zooming, rescaling and shearing. Rotation was performed between 0 and 180°, zooming up to 90% in both directions, rescaling between 93% and 100% and shearing intensity up to 5%.

In the U-Net, each layer in the contracting path is composed of a cascade of double 3x3 convolutions followed by a hyperbolic tangent non-linearity and a 2x2 max-pooling. Similarly, for the expanding path where the max-pooling is replaced by a 2x2 transposed-convolution. The number of feature maps is doubled after each layer in the contracting path and halved in the expanding path. At the final layer a 1x1 convolution with linear output is applied to reduce the number of feature maps to the number of classes (ex: tumor vs. non-tumor).

The network input contains three normalized 2D patches from the three sequences (FIESTA, T2, T1c; total number of sequences ch = 3), which are fed as channels. Coronal and sagittal patches (sized 72x96) were up-sampled to 96x96 to satisfy the U-Net input shape constraint and to train all the three planes simultaneously. Target labels were mapped to binary outputs (0 for background and 1 for tumor/retinal detachment). The initial weights were drawn from Xavier initialization^3^ which is suited for the hyperbolic tangent^4^. The choice of this activation function relies on its stability. We used a weighted cross-entropy as a loss function. Before its computation, a sigmoid is applied to the network output to map it to a probability distribution. The uneven penalization is intended to foster the minority/positive class prediction by a factor of 10 that is approximately the average inverse fraction of the minority class. Minimization was performed using ADAM optimizer^5^ with an initial learning rate of 5e-6 reduced by 10% every 2000 steps (3 to 4 epochs). Tumor and retinal detachment networks were trained on 2D slices containing solely a tumor or a retinal detachment respectively, and were trained for 200 and 130 epochs. Implementation was performed on the framework of TensorFlow and an NVIDIA GeForce RTX 2080 GPU was deployed for training.

**REFERENCES**

1. de Graaf, P. *et al.* Automated segmentation of eye structures and retinoblastoma on MRI using Unet with statistical shape priors. in *ECR 2019, Vienna, AUSTRIA* (2019). doi:10.1371/journal.pone.0173900.

2. Ronneberger, O., Fischer, P. & Brox, T. U-net: Convolutional networks for biomedical image segmentation. in *Lecture Notes in Computer Science (including subseries Lecture Notes in Artificial Intelligence and Lecture Notes in Bioinformatics)* (2015). doi:10.1007/978-3-319-24574-4_28.

3. Glorot, X. & Bengio, Y. Understanding the difficulty of training deep feedforward neural networks. in *Journal of Machine Learning Research* (2010).

4. Kumar, S. K. On weight initialization in deep neural networks. *arXiv* (2017).

5. Kingma, D. P. & Ba, J. Adam: A Method for Stochastic Optimization. in *3rd International Conference of Learning Representations (ICLR)* (2015).

6. Hunter, J. D. Matplotlib: A 2D Graphics Environment, Computing in Science & Engineering. *Comput. Sci. Eng.* (2007).

**SUPPLEMENTARY FIGURES**


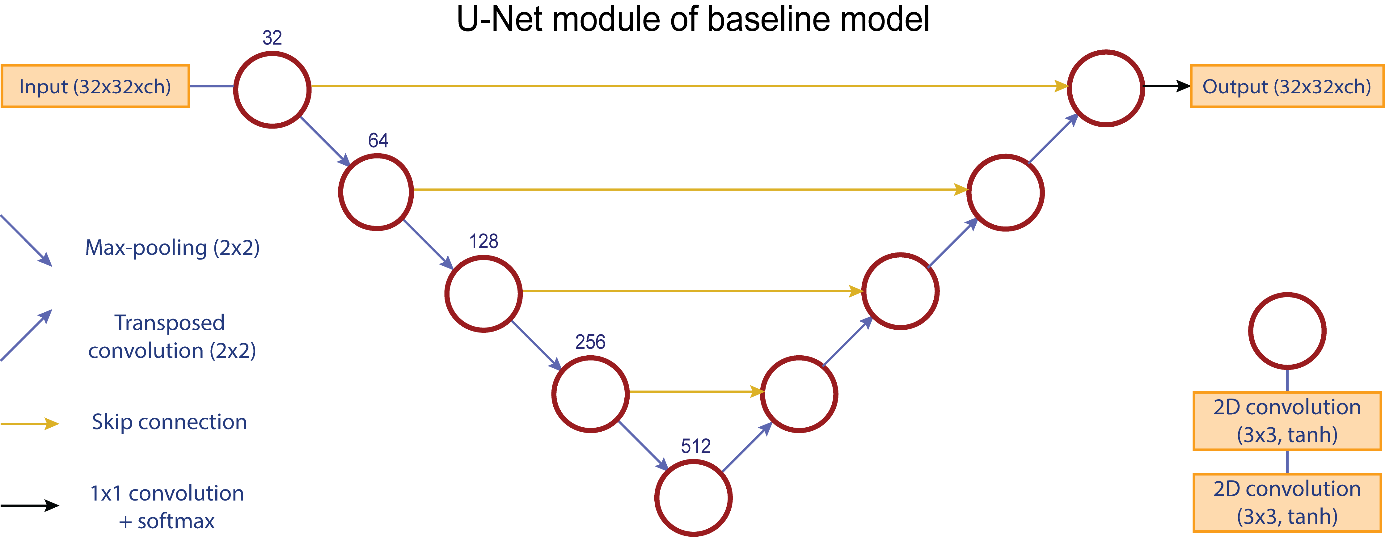


**SUPPLEMENTARY FIGURE S1** Schematic representation of the U-Net used to segment tumor and retinal detachment in the baseline model. Each block consisted of two 3x3 convolutions which were followed by a non-linear activation function. The number on top of network nodes denote the filter number used. Abbreviations: tanh: hyperbolic tangent; h: image channel. Figure was generated with Adobe Illustrator (version 16.0.0; https://www.adobe.com/creativecloud.html).

**
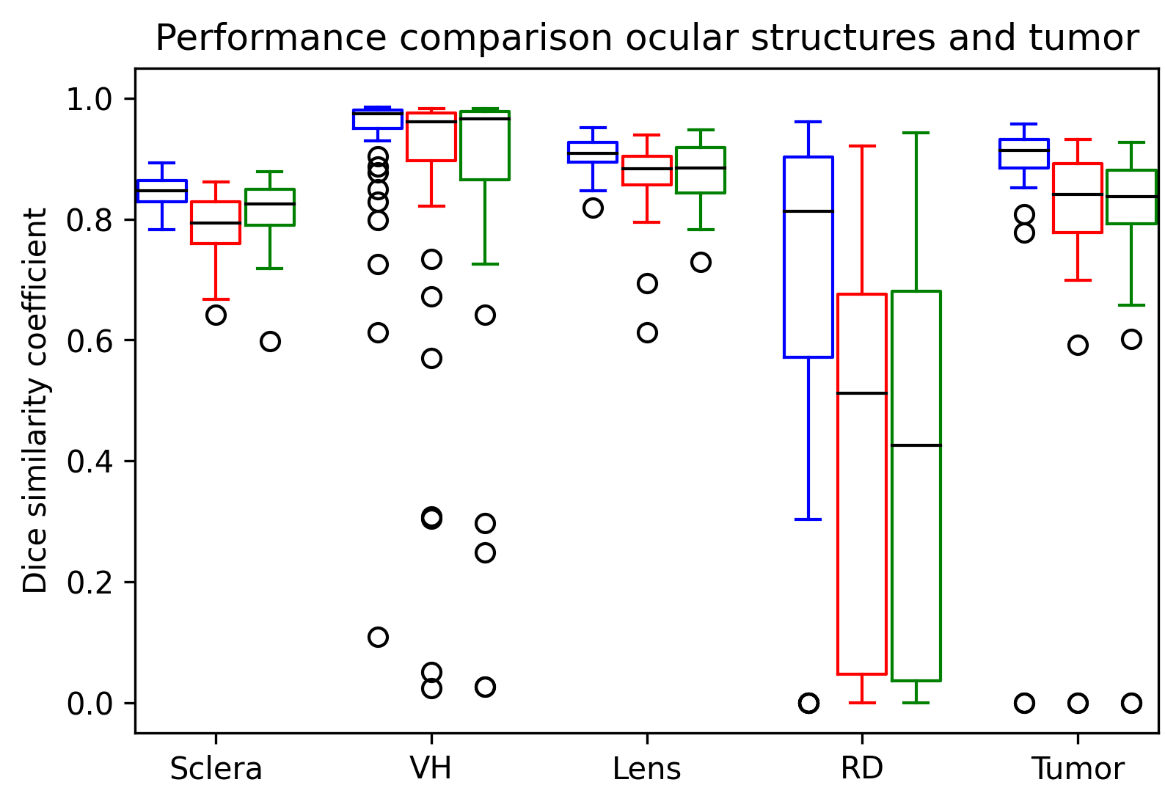
**

**SUPPLEMENTARY FIGURE S2** Ocular structure and tumor segmentation spatial performance comparison for multi-view (blue), axial-view (red) and 3D-view (green) CNNs. Abbreviations: RD: retinal detachment; CNN: convolutional neural network. Figure was generated using Python (version 3.6.9; <https://www.python.org>) including the package Matplotlib (version 3.3.1; <http://matplotlib.org>)^6^.

**
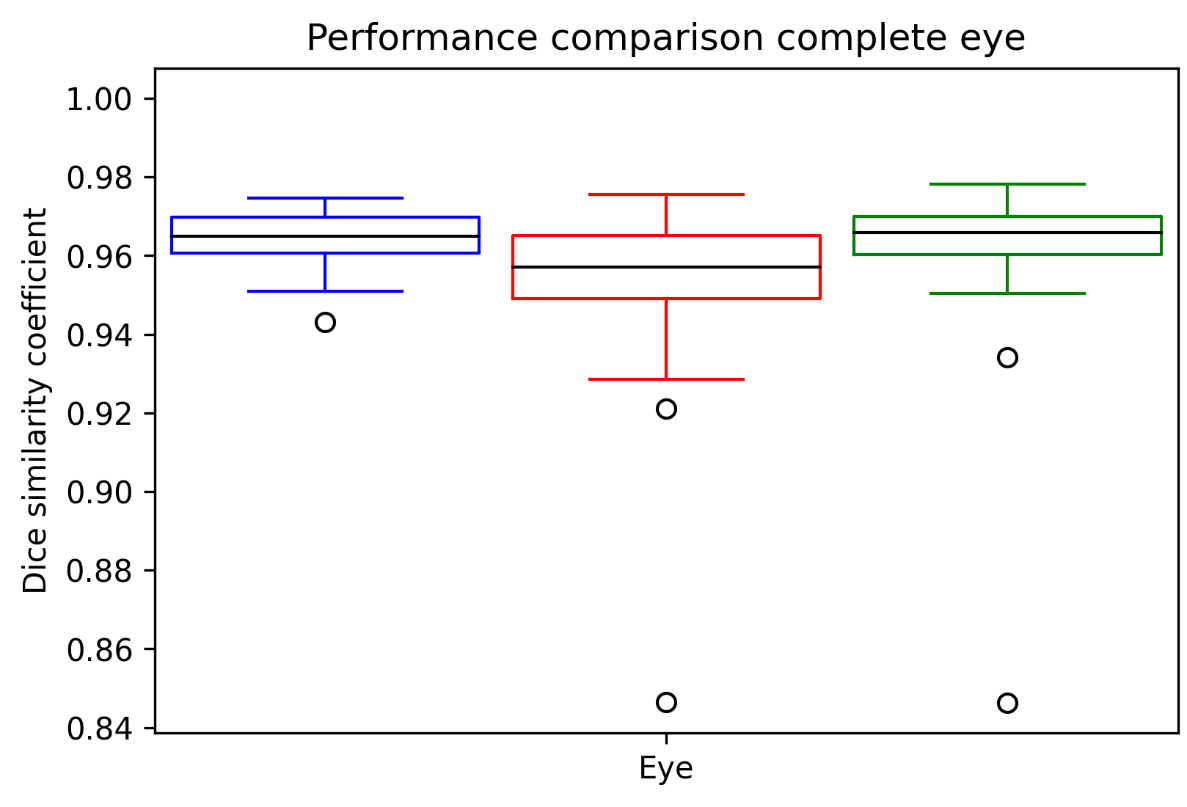

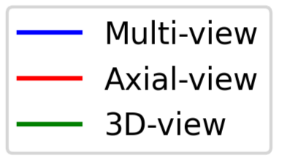
**

**SUPPLEMENTARY FIGURE S3** Complete eye segmentation spatial performance comparison for multi-view (blue), axial-view (red) and 3D-view (green) CNNs. Abbreviations: RD: retinal detachment; CNN: convolutional neural network. Figure was generated using Python (version 3.6.9; <https://www.python.org>) including the package Matplotlib (version 3.3.1; <http://matplotlib.org>)^6^.

**SUPPLEMENTARY TABLES**

**Baseline and MV-CNN sub-model performances**

|  | **Structure** | **Baseline** | **MV-CNN** |  |  |  |
| --- | --- | --- | --- | --- | --- | --- |
| **Sub-model** |  |  | **T2**  **Multi-scale**  **Flip** | **T2**  **Multi-scale**  **-** | **T2**  **-**  **Flip** | **T2**  **-**  **-** |
| **ICC** | **Eye** | 0.919 | **0.997** | **0.997** | 0.995 | 0.994 |
|  | **Tumor** | 0.688 | 0.995 | **0.996** | 0.993 | 0.992 |
| **DSC** | **Eye** | 0.949±0.015 | 0.961±0.007 | **0.965**±0.006 | 0.958±0.013 | 0.960±0.020 |
|  | **Sclera** | 0.669±0.053 | 0.822±0.032 | **0.843**±0.026 | 0.810±0.038 | 0.836±0.036 |
|  | **VH** | 0.792±0.216 | 0.920±0.135 | 0.925±0.143 | 0.893±0.188 | 0.910±0.163 |
|  | **Lens** | **0.935**±0.021 | 0.898±0.030 | 0.908±0.029 | 0.887±0.037 | 0.897±0.037 |
|  | **RD** | 0.502±0.267 | 0.745±0.185 | **0.792**±0.167 | 0.688±0.263 | 0.721±0.232 |
|  | **Tumor** | 0.661±0.237 | 0.826±0.230 | **0.845**±0.234 | 0.816±0.228 | 0.830±0.231 |
|  |  |  |  |  |  |  |
|  | **Structure** | **Baseline** | **MV-CNN** |  |  |  |
| **Sub-model** |  | **-** | **-**  **Multi-scale**  **Flip** | **-**  **Multi-scale**  **-** | **-**  **-**  **Flip** | **-**  **-**  **-** |
| **ICC** | **Eye** | **-** | **0.997** | 0.996 | 0.990 | 0.986 |
|  | **Tumor** | **-** | 0.994 | 0.993 | 0.978 | 0.974 |
| **DSC** | **Eye** | **-** | 0.964±0.007 | 0.961±0.008 | 0.953±0.020 | 0.953±0.019 |
|  | **Sclera** | **-** | 0.841±0.026 | 0.835±0.031 | 0.820±0.042 | 0.821±0.043 |
|  | **VH** | **-** | 0.929±0.132 | **0.931**±0.135 | 0.896±0.188 | 0.903±0.178 |
|  | **Lens** | **-** | 0.910±0.033 | 0.907±0.030 | 0.895±0.032 | 0.898±0.042 |
|  | **RD** | **-** | 0.758±0.020 | 0.782±0.178 | 0.662±0.284 | 0.696±0.260 |
|  | **Tumor** | **-** | 0.841±0.233 | 0.841±0.234 | 0.794±0.225 | 0.805±0.226 |

**SUPPLEMENTARY TABLE S1** Overview of volumetric and spatial performances of the baseline and multi-view convolutional neural network models. The highest mean values of each row are depicted in bold. Abbreviations: ICC: intra-class correlation; DSC: Dice’s Similarity Coefficient; MV: multi-view; CNN: convolutional neural network; VH: vitreous humour; RD: retinal detachment.
